# Gestational Thrombocytopenia: A Review on Recent Updates

**DOI:** 10.7759/cureus.23204

**Published:** 2022-03-16

**Authors:** Elmukhtar Habas, Amnna Rayani, Gamal Alfitori, Gamal Eldin Ahmed, Abdel-Naser Y Elzouki

**Affiliations:** 1 Internal Medicine, Hamad Medical Corporation, Doha, QAT; 2 Internal Medicine, Hamad General Hospital, Doha, QAT; 3 Hematology/Oncology, Tripoli Pediatric Hospital, Tripoli University, Tripoli, LBY; 4 Obstetrics and Gynecology, Woman Health and Research Center, Doha, QAT

**Keywords:** single-baby pregnancy, placenta, itp, pregnancy-induced thrombocytopenia, gt, gestational thrombocytopenia

## Abstract

Thrombocytopenia is a condition in which the blood platelet count is low. It is well established that the mild thrombocytopenia frequency is higher in normal pregnancy. This type of thrombocytopenia was named pregnancy-induced thrombocytopenia. However, recently, it has been widely known as gestational thrombocytopenia (GT). The rate is higher in women with a prior GT history and multiple pregnancies. However, it appears that GT is a physiological response to the pregnancy; placenta's peculiar structure and its unique blood flow pattern play major roles in GT development. There are no specific, precise, or known underlying pathophysiological mechanisms of GT, and no new specific management strategies are published yet. Therefore, we decided to do a non-systematic review of any recent updates that had been published in PubMed, EMBASE, and Web of Science about the pathophysiology of GT, its treatment, and other related topics.

## Introduction and background

A thrombocyte count of <150 × 10^3^/µL is considered thrombocytopenia in the general population. However, a platelet count of >116 × 10^3^/μL is the normal average thrombocyte count in a pregnant female. Thrombocytopenia occurs in 5%-10% of pregnancies and during the postpartum period [1]. A study found that pregnancy-induced thrombocytopenia affects up to 12% of pregnancies [2]. Gestational thrombocytopenia (GT) is frequently found accidentally in investigations during pregnancy assessment visits. GT is also known as incidental thrombocytopenia of gestation (pregnancy-induced thrombocytopenia). GT was reported to cause low platelets in approximately 75% of thrombocytopenic pregnant females [3]. It is often mild and commonly observed in the third trimester, presenting without significant symptoms or clinical signs [4,5].

Excluding other secondary causes of low platelets, such as idiopathic thrombocytopenia (ITP), systemic lupus erythematosus (SLE), pseudothrombocytopenia, and other rare causes, is needed before diagnosing GT. The most frequent etiology of a moderate to severe low platelet count reported by Kim et al. was ITP (42.3%), followed by GT (34.6%) [6]. ITP usually presents with significantly lower platelet counts than GT (52.4 × 10^3^/μL vs. 80.5 × 10^3^/μL, P=0.041) [6]. Another study reported a moderate to severe low platelet count (platelet count <100 × 10^3^/μL) occuring due to GT (59%), preeclampsia (22%) with severe features of HELLP (hemolysis, elevated liver enzymes, and low platelet count) syndrome, and ITP (11%). Antiphospholipid syndrome, disseminated intravascular coagulation (DIC), dilutional thrombocytopenia, and myeloproliferative neoplasm were the cause in 8% of thrombocytopenia cases during pregnancy [4].

GT is a self-limiting benign condition commonly observed in the third trimester with no adverse outcomes and requires no extra assessment or intervention [7]. GT improves rapidly in the postpartum period in those who had not had any prior history of low platelet count before pregnancy [8]. During pregnancy, severe thrombocytopenia (<100 × 10^3^/µL or 70 × 10^3^/µL) is not typically linked with GT; hence, other causes must be ruled out (Table 1) [9,10].

**Table 1:** Causes of low platelets during gestation

Common causes	Rare causes
Gestational thrombocytopenia	Systemic lupus erythematosus
Severe preeclampsia	Thrombotic thrombocytopenia
Immune thrombocytopenia	Hemolytic uremic syndrome
HELLP (hemolysis, elevated liver enzymes, and low platelets) syndrome	Hematological diseases (e.g., lymphoma, leukemia, etc.)
Disseminated intravascular coagulation	Folic acid deficiency
Drugs (heparin)	Type 2B von Willebrand’s disease
	Congenital thrombocytopenia
	Viral infection (HIV, glandular fever)

Hypertensive disorders that can cause low platelets during pregnancy include preeclampsia and HELLP syndrome. Sainio et al. reported that preeclampsia (16%) and ITP (3%) are the causes of thrombocytopenia [11]. Thrombocytopenia severity generally parallels the underlying preeclampsia. Thrombocytopenia may occur before the other preeclampsia manifestations during the late second or early third trimester of pregnancy [10]. In preeclampsia, the low platelet count is primarily due to microangiopathy disease [12]. On the other hand, thrombocytopenia pathophysiology due to HELLP syndrome has exceptional characteristics [13].

## Review

Method

Literature search strategies were conducted using Medical Subject Headings (MeSH) and text keywords related to gestational and pregnancy-induced thrombocytopenia. The literature search was performed using PubMed, EMBASE, and Web of Science.

Gestational thrombocytopenia

GT is described as thrombocyte counts of <150 × 10^3^/μL in gravida females [14]. GT appears in 4.4%-11.6% of pregnancies, representing about 75% of the thrombocytopenia cases during pregnancy [5,15]. Low platelets may be caused by various conditions connected to physiological changes during pregnancy [14]. GT occurs more with multiple gestations than single-baby pregnancies, and is 14-fold higher in women with a previous GT history [16,17]. Shin et al. reported that thrombocytopenia during pregnancy is caused mainly by GT (>70%), preeclampsia (21%), and ITP (3%), with other causes accounting for only 6% cases [18]. It is noticeable that the thrombocyte count decreases at the beginning of the second trimester of the pregnancy, and it may continue even after delivery [1]. However, Reese et al. reported that platelet counts during uncomplicated pregnancies did not support this concept [9]. The reduction in platelet counts might be due to the increased fluid retention, promoted platelet clearance rate due to the increased platelet volume and width, and high platelet-derived cyclooxygenase products. Furthermore, the thromboxane-A2 concentration rises significantly during the second and third trimesters, increasing the thrombus formation rate, platelet destruction, and consequently thrombocytopenia. Women with severe GT, decreased serum anti-thrombin-III, and HELLP syndrome have a higher risk of low platelet count recurrence during subsequent pregnancies [19].

The generally accepted classification describes GT as mild (100-150 × 10^3^/μL), moderate (50-100 × 10^3^/μL), and severe thrombocytopenia (<50 × 10^3^/μL). Mild thrombocytopenia occurs in 7.6% of pregnancies, and >70% of these women have no other known medical conditions that cause the low platelet count. It is highly recommended that a pregnant woman with a platelet count <100 × 10^3^/μL should be evaluated clinically and thoroughly assessed for any other possible underlying medical causes of thrombocytopenia before labeling her as a GT patient (Table 1). The typical features of GT are summarized in Table 2.

**Table 2:** Characteristic features, diagnosis, and prognosis of gestational thrombocytopenia

Clinical features
Mild thrombocytopenia (in 99% of women)
Platelet count ≥70 x 10^3^/μL
No increased bleeding or bruising
No associated abnormalities on complete blood count
No fetal or neonatal thrombocytopenia
Diagnosis
It is a diagnosis of exclusion; no diagnostic testing is required
A history of mild thrombocytopenia during a previous pregnancy supports the diagnosis
Prognosis
GT resolves postpartum; usually the platelet count returns to normal within 6 weeks
A benign, self-limited condition that requires no additional evaluation or treatment

Pathophysiology of GT

The pathophysiology of GT is not clearly known, although two possible reasons are speculated. Increased thrombocyte activation happens at placental circulation, causing substantial platelet consumption. Furthermore, it appears that a physiologic adjustment that occurs during pregnancy is another contributory factor. Increased plasma volume and fluid retention, placenta blood pooling, and increased platelet consumption by the placenta are the leading factors for the development of GT [19]. Furthermore, other additional physiologic alterations associated with normal pregnancies include increased blood pressure (~20%) that may cause thrombocytopenia. Moreover, it was reported that the architecture of placenta is similar to the architecture of spleen and liver, increasing platelet trapping and sequestration, causing GT [19].

Placental structure and blood flow

Blood circulates through the placenta directly from arterioles to venules bypassing the capillaries, and some of the circulating blood shunts to lower pressure of intervillous spaces of the placenta. The same blood flow pattern is noted in the liver and spleen, leading to the destruction of platelets. This similarity of placental blood flow has led to the conclusion that GT might be due to platelet sequestration in the placenta [19]. Furthermore, twins or multiple pregnancies have a higher risk of developing GT due to a big-sized placenta or the presence of more than one placenta, supporting the placenta’s role in GT pathogenesis and severity [20].

Pseudothrombocytopenia

Hemodilution occurs in normal pregnancies leading to low platelets (pseudothrombocytopenia). In pseudothrombocytopenia, the platelet count appears low, although the platelet count is normal. Pseudothrombocytopenia has been progressively designated in patients having various disorders, including coronavirus disease 2019 (COVID-19) infection, mainly due to inappropriate antidiuretic hormone secretion (ADH) secretion [21]. Increased blood cell aggregation is a physiological change that may lead to thrombocytopenia [22].

Pseudothrombocytopenia occurs due to iatrogenic causes such as antithrombotic agents. It was reported that pseudothrombocytopenia is related to the platelet counting method and prevention of clots by ethylenediaminetetraacetic acid (EDTA), sodium citrate, or heparin in the test tube. Reporting the method of platelet counting used is essential to decrease the overdiagnosis of pseudothrombocytopenia [22].

Accurate diagnosis of pseudothrombocytopenia has a clinical impact to avoid unnecessary interventions such as platelet transfusion, splenectomy, and bone marrow aspiration and biopsy [23]. Therefore, using the following major criteria is needed to avoid pseudothrombocytopenia diagnosis: (a) platelet count <100 × 10^3^/μL, (b) no clinical features of bleeding diathesis, (c) existence of clumps in EDTA samples, and (d) time-dependent fall of platelet count [24]. Iatrogenic and artefactual laboratory thrombocytopenia in pregnancy must be excluded before any intervention. However, a peripheral blood smear sometimes is required to rule out hemolysis. Moreover, multidisciplinary management must be conducted before going for the aforementioned interventions [23].

Management of GT

There is no intervention required for GT before or during pregnancy, although the recurrence rate is high for the previously affected females. During the antenatal period, usually, there is no specific therapy. However, regular platelet count monitoring is advisable every four weeks. Closer to the third trimester or if platelets reach clinically significantly low levels, it would be advisable to repeat the platelet count every one to two weeks.

Vaginal delivery is expected in cases of GT, and the cesarean section is reserved for obstetric indications rather than thrombocytopenia. Furthermore, both vacuum and forceps-assisted vaginal delivery can be safely conducted in GT women with a thrombocyte count of >20 × 10^3^/μL [25]. However, when platelet counts are too low (<20 × 10^3^/μL), it is advisable to avoid instrumental-assisted delivery, minimizing the risk of hematoma for the baby and the mother.

There are some concerns related to epidural analgesia and anesthesia with a risk of epidural hematoma in patients with hypertension and positive anticardiolipin antibodies. Other anesthesia physicians do not recommend regional anesthesia, particularly in patients with a platelet count <100 × 10^3^/μL. Reports of pregnant women with unknown or unexplained thrombocytopenia had noted different safe values of platelet counts regarding regional anesthesia [26-28]. It was reported that regional anesthesia could be safely conducted in a pregnant woman with a thrombocyte count of 50-79 × 10^3^/μL [29]. The study suggested that in a non-preeclamptic pregnant woman who has stable thrombocyte counts and no clinical evidence of bleeding, regional anesthesia can be performed as long as the platelet count is >50 × 10^3^/μL [28]. Others reported that patients with thrombocyte counts of 50-80 × 10^3^/μL and 20-40 × 10^3^/μL could safely have epidural anesthesia and lumbar puncture [29-31]. van Veen et al. reported that lower platelet counts appear to be safe, although there are not enough published data to establish these recommendations for a lower platelet count at that stage. Females with a thrombocyte count of 50-80 × 10^3^/μL required either epidural or spinal anesthesia, and women with a thrombocyte count of 20-40 × 10^3^/μL required lumbar puncture; both procedures were conducted without significant bleeding or hematoma development. However, personal decision constructed on the calculation of risks and benefits must be considered [32]. Some anesthesiologists do not advise regional anesthesia in women with significant thrombocytopenia and still insist on treating each case based on the woman's status. Therefore, convincing them to change their recommendations requires performing new larger scale studies.

The bleeding threshold varies between patients and differs according to thrombocytopenic disorders [23]. It was reported that moderate thrombocytopenia is not accompanied by a higher risk of bleeding, a need for blood transfusion, or increased risk of adverse events that occur after cesarean section if the other risks of bleeding are eliminated [33]. The best evidence suggests that the risk of hematoma is unlikely with a platelet count >70 × 10^3^/µL, according to a 2021 Society for Obstetric Anesthesia consensus [34]. Furthermore, many women had epidural anesthesia and were thrombocytopenic without significant complications, although knowing the platelet count is highly advisable [23]. In patients with severe thrombocytopenia, discussing neuraxial analgesia or other anesthesia options with the anesthesia physician before delivery is essential. Additionally, obstetricians' awareness about the local recommendation and preferences of the anesthesiologist(s) performing the neuraxial procedure is advisable.

Pregnancy outcome in GT

There is no significant harmful pathology reported for the baby and the mother due to GT [35]. In a study of 162 women with thrombocytopenia, 74 of them had GT; no infant was born with a platelet count <50 × 10^3^/μL or intracranial hemorrhage [36]. Habas et al. reported lower mean platelet counts (41 ×10^3^/μL) in hypertensive GT pregnant women during the third trimester. The same study concluded that the pregnancy proceeded safely without complications for the babies and mothers [37]. Another report noted that GT was an uneventful condition for the participants except for one woman with a platelet count of <50 × 10^3^/μL who had one child with a trisomy-21 anomaly. The study concluded that the trisomy was not related to thrombocytopenia [38]. Although babies of GT mothers are usually safe and are unlikely to have complications following delivery, all babies must have a platelet count check-up on the first day of delivery and the fourth day, especially if the maternal platelet count was <80 × 10^3^/μL [15].

## Conclusions

Thrombocytopenia in pregnant women is commonly due to GT, although other causes such as pseudothrombocytopenia must be excluded first. GT does not impose any fetal or maternal risk, and it is a self-limited benign condition, requiring no additional work-up or therapy in most cases. GT improves following delivery or during the postpartum period. However, the normalization of platelet count may need more than six weeks in some cases. A history of a mild low platelet count that improves between pregnancies strongly indicates GT's diagnosis, especially when the platelet count is ≥70 × 10^3^/μL. No confirmatory investigations are considered diagnostic for GT or differentiate between GT and ITP. Vaginal delivery is a safe procedure in GT, and a cesarean section should be offered when there are obstetric indications.
